# Development and validation of prognostic nomogram in patients with nonmetastatic malignant melanoma: a SEER population‐based study

**DOI:** 10.1002/cam4.3318

**Published:** 2020-09-17

**Authors:** Yu Xiao, Shanshan Peng, Youhong Hu, Jie Zhang, Xianwei Cao

**Affiliations:** ^1^ The Central Hospital of Xiaogan Xiaogan Hubei China; ^2^ The First Affiliated Hospital Of Nanchang University Nanchang Jiangxi China

**Keywords:** nomogram, seer, prognosis, survival

## Abstract

**Background:**

The condition of tumor recurrence and overall death can be worried in the progress of nonmetastatic malignant melanoma (NMMM). Our goal was to construct and validate a prognostic nomogram from a large population database, which is vital for physicians to predict the 3‐ and 5‐year overall survival (OS) rates of patients with NMMM.

**Methods:**

According to the Surveillance, Epidemiology, and End Results (SEER) program, patients were collected and randomly assigned into the training and validation cohorts. Several independent risk factors were identified based on the methods of univariable and multivariable cox hazards regression and were incorporated to develop a nomogram. The concordance index (C‐index), the area under the receiver operating characteristics (AUC) curve and calibration plot were confirmed to assess predictive power of the nomogram. Decision curve analysis (DCA) was performed to measure nomogram for the clinical practice.

**Results:**

A total of 66192 eligible patients, randomly assigned into 70% of training (n = 46 336) and 30% of validation cohorts (n = 19 856), were selected in this study. The selected independent factors were applied to develop a nomogram, and validated indexes indicated nomogram had a good discrimination ability. The C‐index for OS rates was 0.817 (95% CI: 0.811‐0.823) in training cohort and 0.817 (95% CI: 0.809‐0.825) in validation cohort, respectively. The AUCs of 3‐ and 5‐year OS rates were more than 0.79, and the calibration plots also showed a good power for the nomogram. DCA demonstrated that constructed nomogram can provide clinical net benefit.

**Conclusion:**

We constructed a novel nomogram that more accurately and comprehensively predict OS with nonmetastatic malignant melanoma patients, which is vital for clinician to improve individual treatment, make reasonable clinical decisions, and set appropriate follow‐up strategies.

## Graphical abstract

we constructed and validated a nomogram based on a large population database, which will predict the 3‐and 5‐year overall survival rates of patients with non‐metastatic malignant melanoma.

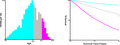

## 1 INTRODUCTION

Cutaneous malignant melanoma (CMM) is a highly malignant tumor, ranking fifth among the most common cancers in men and seventh in women ^1^. The incidence and mortality of CMM continues to rise annually.^2^, ^3^ In USA, melanoma is the fifth most common malignant tumor and its incidence was rapidly increasing 96 480 new cases in 2019.^3^ Although most localized CMMs had a high 5‐year survival rate, one‐third of CMM patients may experience disease recurrence and a range of 10%‐40% patients made a diagnosis with localized lesions die from CMM eventually.^4^, ^5^ Therefore, it is particularly significant to identify and monitor patients who have already suffered from CMM in order to detect the prognosis of cancer as early as possible.

There is about 90% of melanomas diagnosed as primary cancers, and all of them did not found any evidence of metastasis. The 10‐year caused survival rate of cancer is 75%‐95%.^3^, ^6^ Histologically, the most important prognosis factors for primary melanoma with nonmetastases, as reported in previous studies, are: breslow's depth, ulceration, mitotic rate, treatment.^7^, ^8^, ^9^, ^10^, ^11^, ^12^, ^13^, ^14^, ^15^ The poor independent factors also include age, sex, race, marital status, and anatomic site, as well as American Joint Committee Cancer (AJCC) stage provided a rough prediction for estimating the development of cancer and for the selection of making proper clinical decision.^13^ But, it is insufficient for clinical to predict the personalized prognostic result. In this study, we identified those independent factors from the SEER program, which can provide more people benefit from our study.

Nomogram is a visual calculation that incorporates several independent‐related variables to predict a survival rate or risk of disease, which mainly depends on traditional statistical methods including cox regression or logistical regression.^16^, ^17^ To date, nomogram is widely used to help physician accurately and timely estimate the prognosis of patients.^15^, ^17^ Previous several studies have reported that primary clinical features were correlated with prognosis of patients with CMMs,^15^, ^18^, ^19^ but few studies constructed a nomogram, based on those common clinical features, to predict the survival rate of patients with NMMM.^19^ Therefore, our purpose of the present study was to identify clinicopathological factors associated with prognosis basing on the data from SEER database. In particular, we sought to construct and validate a nomogram for predicting the individual 3‐ and 5‐year OS rates of patients with NMMM.

## 2 METHODS

### 2.1 Data source and selection of variables

**Figure 1:**
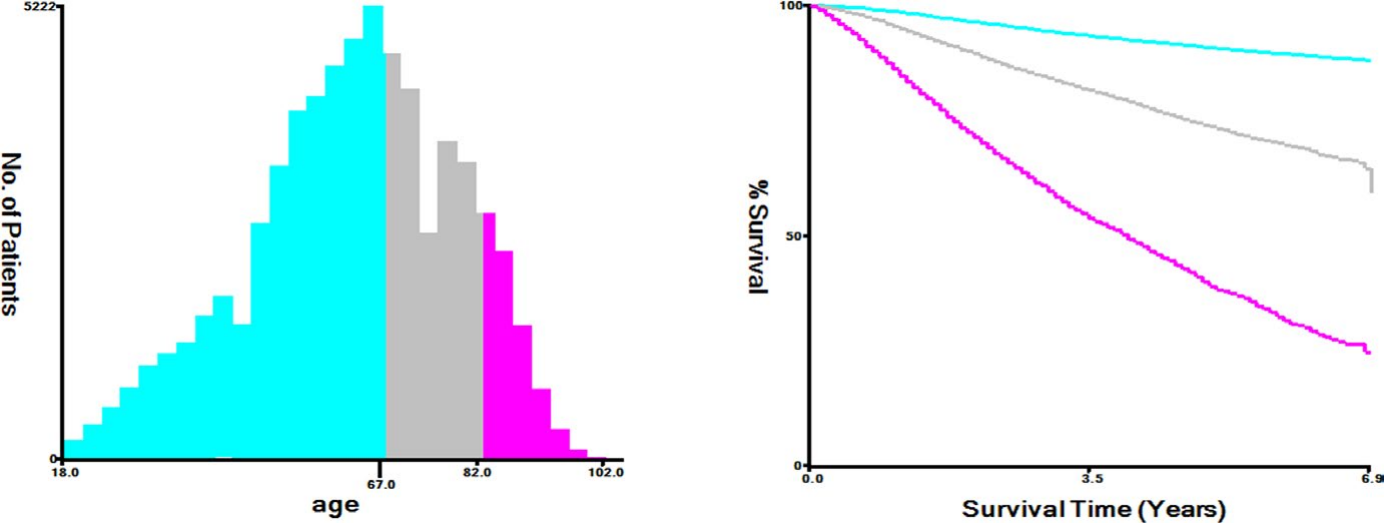
The optimal cut‐off values for age were <67, 67‐82, and >82 years old

The clinical information, including sex, age, race, marital status, anatomic sites, stage, depth, mitoses, ulceration, treatment, survival time, and survival status, were selected from the SEER 18 Regs Research Date, Nov 2018 Sub. The present research from the SEER program was conducted for all patients with nonmetastatic melanoma diagnosed during 2010‐2015. The SEER research data were available using the SEER*Stat 8.3.6 (http://seer.cancer.gov//seerstat/). According to the International Classification of Diseases for Oncology‐O‐3^20^, anatomic sites were classified into five sites as follows: face/ear (C440‐C443), scalp and neck (C444), trunk (C445), extremities (C446‐C447), and NOS/overlapping codes (C448‐C449). The stage of lymph node included localized (confined to primary organ, the skin), regional (spread to surrounding organs or local lymph nodes), and distant (spread to distant lymph nodes). The optimal cutoff values were utilized to convert the continuous variable into the categorical variable with X‐tile software (Yale University, New Haven, Connecticut, USA)^21^ (Figure 1). By age in this study, the optimal cutoff values were subdivided into <67, 67‐82, and> 82 years old. The cutoff values for depth, as reported previous studies, were 0.01‐0.99 mm, 1.00‐2.00 mm, 2.01‐4.00 mm, and >4.00 mm.^6^, ^13^

According to the inclusion and exclusion criteria, 66192 patients with nonmetastatic malignant melanoma were finally collected in our study, For the development and validation of the nomogram, approximately 70% of the patients were classified into a training cohort (n = 46336), and the remaining were categorized as validation cohort (n = 19856). The SEER database agreement was signed and provided a license for accessing the SEER information (accession username:10883‐Nov2019). The SEER database is publicly accessible in the world, as a consequence, we did not provide the approval and informed consent of an institutional review committee in this study.

### 2.2 Statistics analysis

The analysis of descriptive statistics was used in demographic and clinical factors, and the associations between the training cohort and the validation cohort were calculated using chi‐squared test.

The corrections of relevant clinical variables with overall survival were assessed using univariable Cox proportional hazards regression. In training cohort, the method of multivariable Cox proportional hazards regression was conducted to identify variables. Hazard ratios (HRs) were showed with their 95% CIs. The identified independent prognostic factors were integrated to develop a nomogram for predicting the probability of 3‐ and 5‐year OS rates. The discriminating ability of the nomogram was assessed by the concordance index or ROC curve with training cohort and validation cohort, which quantitatively estimates the level of concordance between the predicted rates and the actual probability of having the event of interest. The C‐index of 0.5 suggests the absence of discrimination, whereas a C‐index of 1.0 shows perfect separation of patients with different outcomes. In brief, the higher the C‐index is, the better predictive ability of nomogram. Similarly, the bigger the AUC is, the better accurately predictive ability of nomogram. Meanwhile, the DCA was conducted to determine the clinic value of the predictive model by quantifying the net benefit at disparate threshold probabilities.^22^

In addition, calibration plots were evaluated by comparing the relationship between the observed outcome frequencies and the predicted probabilities, which were performed by bootstrapping with 1000 resamples. In a well‐calibrated model, the predictions are supported to pass through along 45‐degree diagonal line.

All statistical analyses were conducted by software IBM SPSS Statistics 26.0 (SPSS, Inc, Chicago, IL). The nomogram, receiver operating characteristics (ROC) curves, calibration plots, and DCA curves were performed by R version 3.6.3 (http://www.r‐project.org). All analyses were two sided, and *P* value was determined to be statistically significant.

## 3 RESULTS

### 3.1 Demographic and clinicopathological characteristics

A total of 66192 patients with NMMM were obtained in SEER database between 2010 and 2015. In terms of demography, the number of patients in both cohorts mainly was male (59.19%), young (57.23%), married (69.05%), and white (98.70%). And in terms of tumor characteristics, the most primary anatomic site is extremities (44.65%), followed by trunk (32.58%), the most common lymph node stage was localized (88.38%), and the most common depth range of melanoma from 0.01 to 0.99 mm (61.24%). Additionally, most melanoma patients were no ulceration (83.84%) and cell of melan mitose (64.07%), and most patients had a choice of surgery treatment (98.45%). All patient demographic and clinicopathological characteristics are shown in Table 1.

**Table 1:** The demographics and clinical features for NMMM in different cohorts

	Total(%) (n = 66192)	Training cohort(%) (n = 46336)	Validation cohort(%) (n = 19856)	*P* Value
Sex				0.3004
Male	39182 (59.19)	27368 (59.06)	11814 (59.50)	
Female	27010 (40.81)	18968 (40.94)	8042 (40.50)	
Age				0.4341
<67years old	37885 (57.23)	26482 (57.15)	11403 (57.43)	
67‐82 years old	21308 (32.20)	14982 (32.33)	6326 (31.86)	
83‐102 years old	6999 (10.57)	4872 (10.51)	2127 (10.71)	
Marital status				0.0393
Married	45710 (69.05)	31863 (68.77)	13847 (69.74)	
Single/Domestic partner	9843 (14.87)	6974 (15.05)	2869 (14.45)	
Dirvoced and Separated and Widowed	10639 (16.08)	7499 (16.18)	3140 (15.81)	
Race				0.6677
White	65341 (98.70)	45754 (98.74)	19587 (98.64)	
Black	274 (0.42)	191 (0.41)	83 (0.42)	
Asian or pacific islander	422 (0.64)	284 (0.61)	138 (0.70)	
American indian/Alaska native	155 (0.24)	107 (0.24)	48 (0.24)	
Site				0.4202
Face/ears	8657 (13.08)	6055 (13.07)	2602 (13.10)	
Scalp/neck	6248 (9.44)	4417 (9.53)	1831 (9.22)	
Trunk	21573 (32.58)	15020 (32.42)	6553 (33.00)	
Extemities	29561 (44.65)	20732 (44.74)	8829 (44.47)	
NOS/overlapping	153 (0.23)	112 (0.24)	41 (0.21)	
Stage				0.1327
Localized	58502 (88.38)	40958 (88.39)	17544 (88.36)	
Regional	7250 (10.95)	5089 (10.98)	2161 (10.88)	
Ristant	440 (0.67)	289 (0.63)	151 (0.86)	
Depth				0.4554
0.01‐0.99 mm	40535 (61.24)	28431 (61.36)	12104 (60.96)	
1.00‐2.00 mm	12931 (19.53)	8988 (19.40)	3943 (19.86)	
2.01‐4.00 mm	7345 (11.10)	5125 (11.06)	2220 (11.18)	
＞4.00 mm	5381 (8.13)	3792 (8.18)	1589 (8.00)	
Ulceration				0.8088
Absent	55498 (83.84)	38839 (83.82)	16659 (83.90)	
Present	10694 (16.16)	7497 (16.18)	3197 (16.10)	
Mitoses				0.6973
Absent	23782 (35.93)	16626 (35.88)	7156 (36.04)	
Present	42410 (64.07)	29710 (64.12)	12700 (63.96)	
Treatment				0.4921
Non‐surgery	1025 (1.55)	728 (1.57)	297 (1.50)	
Surgery	65167 (98.45)	45608 (98.43)	19559 (98.50)	

Abbreviations: DSW, Dirvoced and Separated and Widowed

### 3.2 Selection of prognostic factors

Univariable analysis showed that age, sex, race, marital status, anatomic site, stage, depth, ulceration, mitoses, and treatment were related to OS. Multivariable analysis indicated that age, sex, race, marital status, anatomic site, stage, depth, ulceration, mitoses, mitoses, and treatment were also verified to be independent prognostic factors for OS. The identified independent prognostic factors are shown Table 2.

**Table 2:** Univariable and multivariate cox analysis for NMMM patients

Variable	Univariable	*P* Value	multivarible	*P* Value
	HR(95%CI)		HR(95%CI)	
Factors selected				
Sex				
Male	Reference	NA	Reference	NA
Female	0.57 (0.54,0.61)	＜.001	0.67 (0.63,0.71)	＜.001
Age				
<67years old	Reference	NA	Reference	NA
67‐82 years old	3.05 (2.87,3.24)	＜.001	2.68 (2.52,2.85)	＜.001
83‐102 years old	9.90 (9.29,10.55)	＜.001	7.10 (6.62,7.60)	＜.001
Marital status				
Married	Reference	NA	Reference	NA
Single/Domestic partner	0.98 (0.91,1.06)	.636	1.26 (1.16,1.36)	＜.001
DSW	2.24 (2.12,2.37)	＜.001	1.43 (1.35,1.52)	＜.001
Race				
White	Reference	NA	Reference	NA
Black	2.72 (2.11,3.50)	＜.001	1.71 (1.32,2.21)	＜.001
Asian or pacific islander	1.39 (1.05,1.85)	.0224	1.12 (0.84,1.49)	.442
American indian/Alaska native	1.46 (0.93,2.28)	.1027	1.31 (0.83,2.05)	.244
Site				
Face/ears	Reference	NA	Reference	NA
Scalp/neck	1.11 (1.02,1.20)	.0171	0.98 (0.90,1.07)	.676
Trunk	0.56 (0.52,0.60)	＜.001	0.89 (0.83,0.96)	.002
Extemities	0.56 (0.52,0.60)	＜.001	0.79 (0.74,0.85)	＜.001
NOS/overlapping	0.91 (0.59,1.40)	.6667	1.18 (0.77,1.81)	.4567
Stage				
Localized	Reference	NA	Reference	NA
Regional	3.62 (3.42,3.82)	＜.001	1.80 (1.69,1.92)	＜.001
Ristant	5.43 (4.52,6.52)	＜.001	3.39 (2.81,4.09)	＜.001
Depth				
0.01‐0.99 mm	Reference	NA	Reference	NA
1.00‐2.00 mm	1.69 (1.57,1.81)	＜.001	1.24 (1.15,1.34)	＜.001
2.01‐4.00 mm	3.57 (3.34,3.81)	＜.001	1.69 (1.56,1.83)	＜.001
＞4.00mm	6.54 (6.41,7.29)	＜.001	2.32 (2.12,2.53)	＜.001
Ulceration				
Absent	Reference	NA	Reference	NA
Present	0.36 (0.31,0.41)	＜.001	1.84 (1.74,1.96)	＜.001
Mitoses				
Absent	Reference	NA	Reference	NA
Present	2.28 (2.15,2.42)	＜.001	1.17 (1.09,1.26)	＜.001
Treatment				
Non‐surgery	Reference	NA	Reference	NA
Surgery	0.36 (0.31,0.41)	＜.001	0.38 (0.33,0.44)	＜.001

Abbreviations: DSW, Dirvoced and Separated and Widowed.

### 3.3 Construction and validation of nomogram

**Figure 2:**
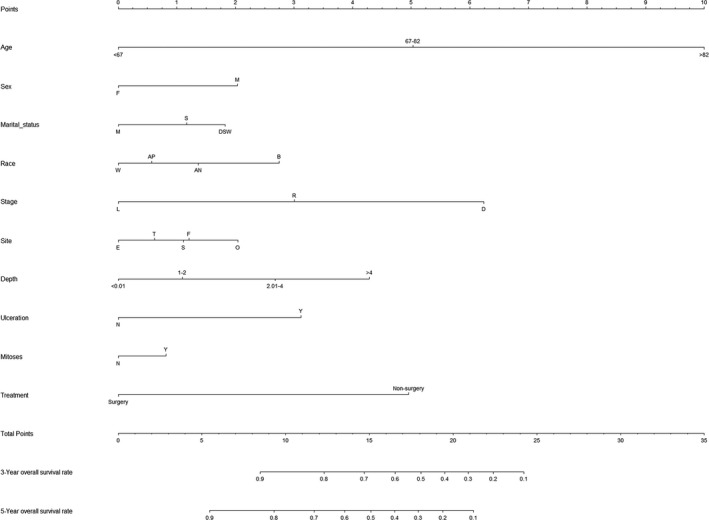
Nomogram for predicting 3‐ and 5‐year overall survival rates of patients with NMMM. F‐female, m‐male; W‐white; B‐black; AP‐Asian or pacific islander; AN‐American indian/Alaska native; L‐localized; R‐regional; D‐ distant; E‐ extremities; F‐face/ears; T‐truck; S‐scalp/neck; O‐NOS/overlapping; N‐absent; Y‐present

**Figure 3:**
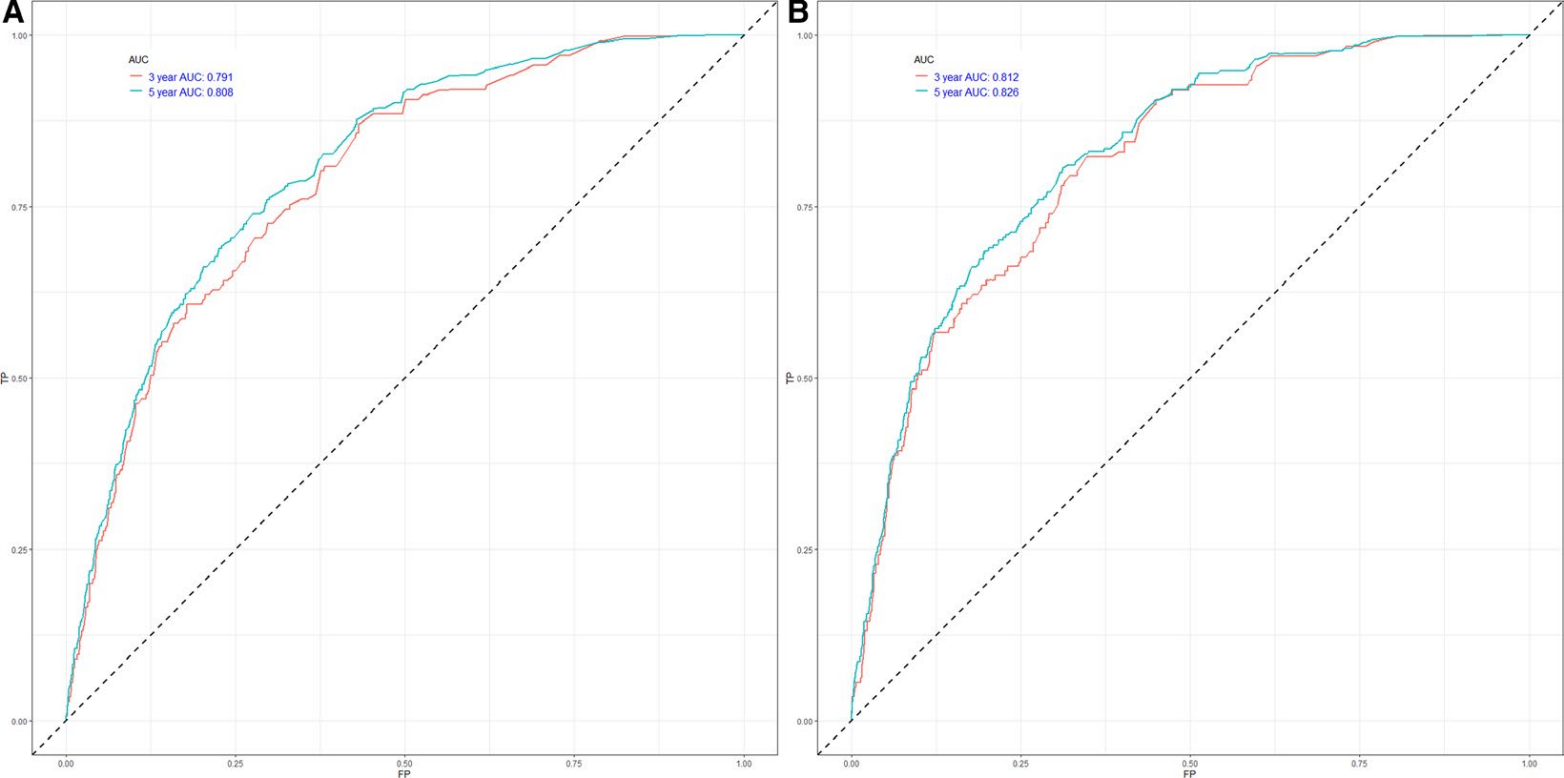
ROC curve analysis to predict 3‐ and 5‐year OS rates in NMMM Patients. (A) ROC curve for the training cohort. (B) ROC curve for the external validation cohort. AUC, area under the curve; ROC, receiver operating characteristic; TP, true positive rates; FP, false positive rate

**Figure 4:**
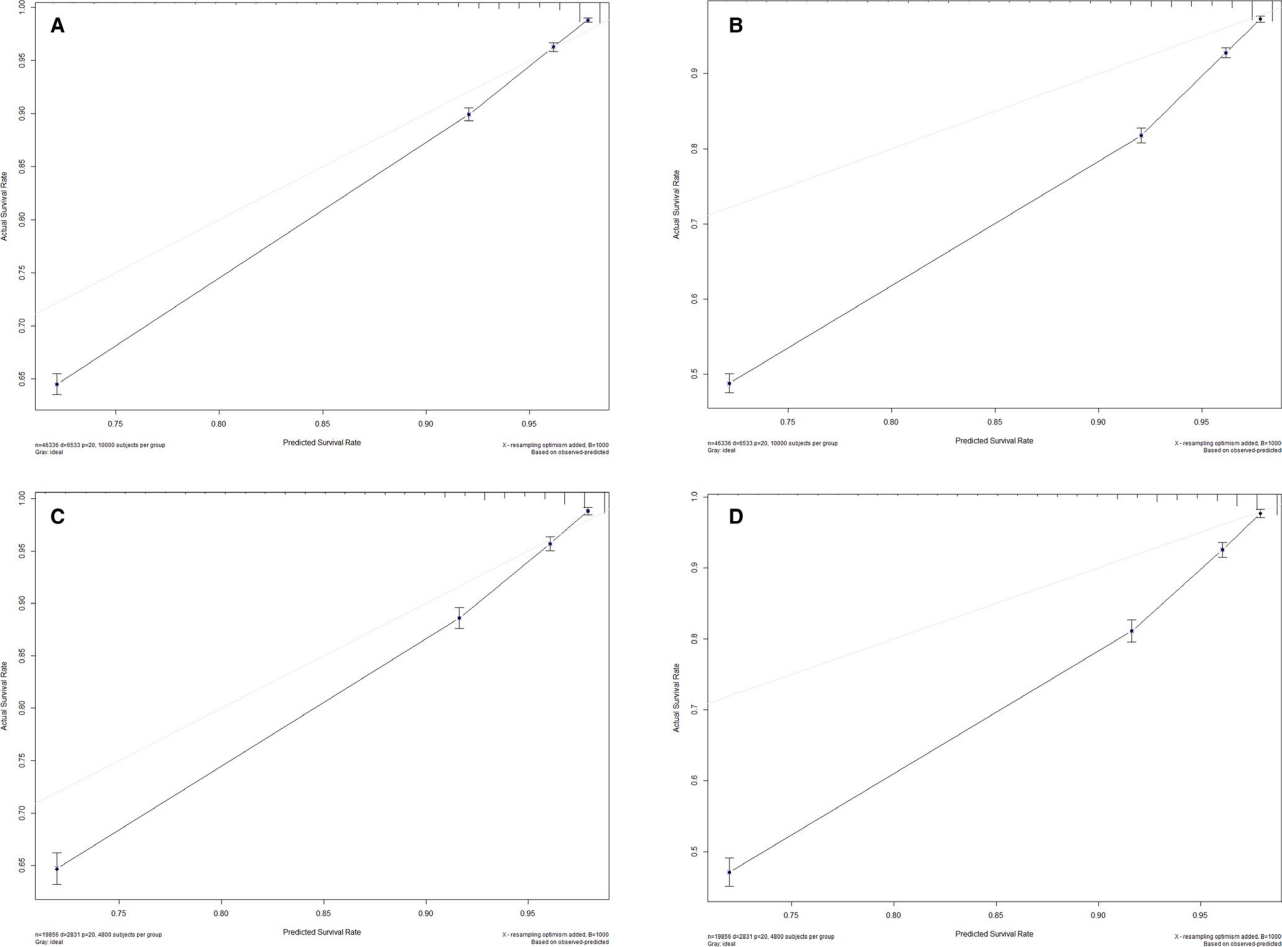
Calibration plots of the nomogram for predicting 3‐ and 5‐year OS rates in NMMM patients, Calibration plots show the relationship between the predicted probabilities base on the nomogram and actual values of the training cohort (A and B) and validation cohort (C and D)

**Figure 5:**
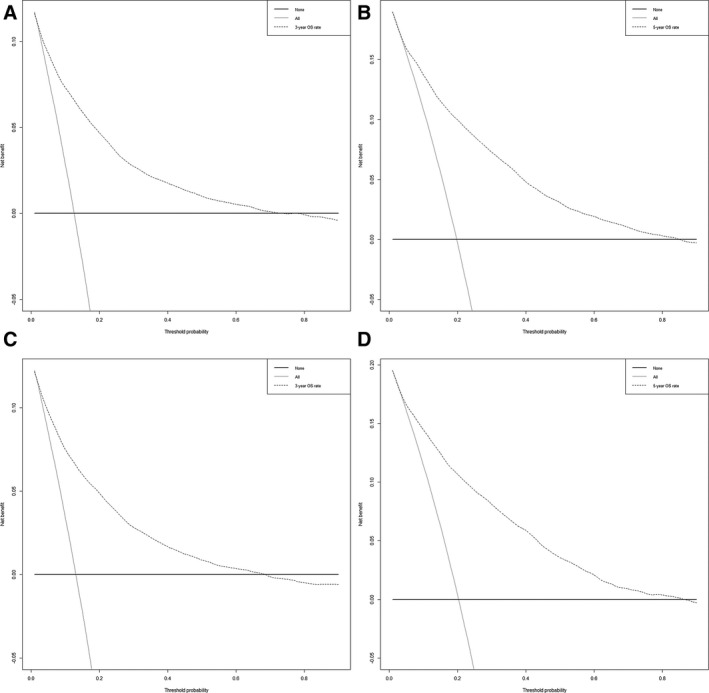
DCA of the 3‐ and 5‐year OS rates for the training and validation cohorts. The abscissa represents the threshold probability and the ordinate represents the net beneft rate. The X‐axis indicates that all samples are negative and all are not treated, with a net beneft of zero. The grey line indicates that all samples are positive. The net benefit is represented by a negative slope. The dotted line does not coincide with the other two lines, and when it is in the upper right corner, it means that the model is valuable. The DCA showed that predicting the 3 and 5‐year OS rates using this nomogram would be better than having all patients or none patients treated by this nomogram with a range of the threshold probability. (A) The DCA of the 3‐year OS rates for the training cohort, a range of the threshold probability between> 1% and < 75%. (B) the DCA of the 5‐year OS rates for training cohort, a range of the threshold probability between> 1% and < 85%. (C) The DCA of 3‐year OS rates for the validation cohort a range of the threshold probability between> 1% and < 68%. (D) The DCA of the 5‐year OS rates for the validation cohort a range of the threshold probability between> 1% and < 86%. DCA, decision curve analysis; OS, overall Survival

The nomogram model was to be provided by incorporating above independent prognostic factors. Higher total points on the basis of the sum of the assigned number of points for each identified factor in the nomogram were connected with a worse prognosis (Figure 2). Internal validation of the training cohort indicated that the C‐index was 0.817 (95% CI: 0.811‐0.823), and the C‐index of the external validation was 0.817 (95% CI: 0.809‐0.825). The high C‐index of internal and external validation indicated a better performance of discrimination. The 3‐ and 5‐year OS rates AUCs of training cohort were 0.791 and 0.808, respectively. Similarly, the corresponding values of the validation cohort were high, 0.812 and 0.826, respectively, which indicated that the normogram had a nice discriminated ability (Figure 3). The calibration curves of internal and external validation demonstrated a consistency between observation and prediction in the probability of 3‐ and 5‐year OS rates, respectively (Figure 4). Moreover, we also performed a DCA, which showed that applying this model would be preferable to having all patients or none patients treated by this model with a range of the threshold probability^22^ (Figure 5).

## 4 DISCUSSION

In our study, we identified that age, sex, race, marital status, anatomic site, stage, depth ulceration, mitoses, and treatment were correlated with prognostic factors in the OS rate of patients with NMMM based on univariable and multivariable cox proportion hazards regression. Meanwhile, we constructed nomogram that quantificationally predicted an individual 3‐ and 5‐year OS rates by patient‐related and tumor‐related factors. This nomogram can be carried out to estimate and inform the prognosis of the patients, as well as to make personalized decisions with regard to the surveillance and therapy.

Several recent studies determined that the advanced age was a poorer prognosis in patients with CMM, indicting age as an independent factor, which was similar to our study, but the cutoff points of the age in different studies were not uniform.^6^, ^7^, ^13^, ^19^, ^23^ Therefore, in present study, the ages were divided into < 67, 67‐82, and> 82 as the cutoff points by X‐tile software, which could be a better tool in distinguishing the survival rate of certain variables ^21^. Meanwhile, we also found that sex was related to prognosis in CMM,^6^, ^7^, ^8^, ^13^, ^19^, ^23^ and female had a higher OS rate, which was consistent with the reported study.^6^, ^7^, ^8^ Non‐married patient, including single and DSW, also showed a worse prognosis in our manuscript, which was line with previous publications.^14^, ^15^ In addition, white individuals were prone to suffer from CMM than other race, as previous studies reported.^1^, ^6^, ^7^, ^8^, ^13^, ^19^ Similarly, our data showed that white patient had a worse prognosis compared to other racial patients. We suspected that ethnic differences affecting the OS rate of patients with CMM may be attributable to the combination of biological effects and epidemiology. Moreover, another reason for the different survival rates of CMM patients may also be different exposure time to the sun light.^2^, ^6^, ^7^, ^13^

For clinicopathological characteristics, previous studies showed that anatomic site was a significant independent factor on OS rates of CMM, and the face/ears in particular had a lower survival rate than other anatomic sites in CMM, which were not in agreement with our results.^8^, ^19^ As the tumor depth, the deeper tumor patients were more likely to have a grim prognosis, which was accordance with our present study.^6^, ^8^, ^9^, ^13^, ^19^ In our study, ulceration and stage of lymph node also were identified as important prognostic factors for affecting the OS rate in patients with CMM, which were in line with other studies.^6^, ^9^, ^13^, ^19^ Abovementioned four variables independent worse prognostic factors may be the result of the close relationship of distant metastases. Notably, mitotic rate often was considered as an independent factors in previous studies.^12^, ^19^ In our study, mitosis was selected as an independent factor by the multivariable cox proportion hazards regression, but mitosis was no longer applied for subclassification of thin melanomas in the eighth AJCC staging system.^13^ The level of invasion was no longer part of the eighth AJCC staging system in reported studies.^6^, ^13^ Therefore, the level of invasion was not enabled into the present study. Additionally, the choice of surgery treatment could improve OS and was a vital protective factor in patients with NMMM, which was consistent with previous research.^24^

Yang et al developed and validated a nomogram for predicting the risk of a cutaneous histopathological subtype of nodular melanoma, and the nomogram was constructed by incorporating several common related factors, including age at diagnosis, sex, marital status, AJCC stage, SEER stage, and lymph node density.^15^ But in our manuscript, we selected common independent risk factors in patients with NMMM as follows: age, sex, race, marital status, anatomic site, stage, depth ulceration, mitoses, and treatment, and these factors were more readily available and comprehensive in clinical work. In addition, our nomogram showed a better discrimination power for predicting prognosis.^15^ To the best of our knowledge, it is the first time that a nomogram has been constructed for effectively predicting the prognosis of patients with NMMM. In the present study, the internal and external C‐index were more than 0.79, showing a delighted discrimination power to provide prognostic information to patients with personalized way. Similarly, the AUCs also suggested that a good discriminated ability. The calibration curves showed that a superior level of consistency on the prediction value for nomogram. In addition, DCA was performed to provide clinical net benefit of predicted model.^22^ In this study, all results showed that the 3‐year and 5‐year OS rates DCA curves for the new model yield significant clinical net benefits.

There were certain limitations in the present study. First, the data performed nomogram came from the SEER database, and the SEER database only contains 27.8% of the U.S. population, therefore, the population and racial were limited. Those factors may be also added in the future predictive model. Second, we did not identify other factors that may affect the prognosis, such as economic conditions, pathological subtype, tumor‐related gene, treatment and complicated disease.^5^, ^6^, ^10^, ^13^ The combination of these information would make the prediction of nomogram more accurate and individual in the future. Finally, patients were consisted of two groups, 70% of them were applied to build and the remaining 30% were conducted to validate the nomogram. The C‐index, AUC, the calibration curve, and DCA performed well, but future studies are necessary in order to externally validate the proposed nomogram.

## 5 CONCLUSION

In summary, we incorporated demographic and clinicopathological characteristics from the SEER database to build an effective nomogram for predicting the prognosis of patients with NMMM. The nomogram could help clinicians more accurately to predict the 3‐ and 5‐year OS rates of individual patient, which will pave the way for follow‐up management measures.

## CONFLICT OF INTEREST

None.

## AUTHOR CONTRIBUTIONS

Conception and design: Yu Xiao, Shanshan Peng, Xianwei Cao. Collection and assembly of data: Yu Xiao, Shanshan Peng, Xianwei Cao, Jie Zhang. Data analysis and interpretation: Yu Xiao, Shanshan Peng, Xianwei Cao, Youhong Hu. Manuscript writing: All authors. Final approval of manuscript: All authors. Accountable for all aspects of the study: All authors.

## Data Availability

The data from present study are available in the Surveillance, Epidemiology, and End Results, https://seer.cancer.gov.
